# Oncologists’ perspective on advance directives, a French national prospective cross-sectional survey – the ADORE study

**DOI:** 10.1186/s12910-024-01046-8

**Published:** 2024-04-10

**Authors:** Amélie Cambriel, Kevin Serey, Adrien Pollina-Bachellerie, Mathilde Cancel, Morgan Michalet, Jacques-Olivier Bay, Carole Bouleuc, Jean-Pierre Lotz, Francois Philippart

**Affiliations:** 1 Department of Anesthesiology and Intensive Care, Saint-Antoine and Tenon Hospitals, Assistance Publique-Hôpitaux de Paris, Paris, France; 2 DMU DREAM, Sorbonne Université, Assistance Publique-Hôpitaux de Paris, GRC 29, Paris, France; 3 Department of Anesthesiology, Perioperative and Pain Medicine, Stanford University School of Medicine, Stanford, CA USA; 4 Department of Anesthesiology and Intensive Care, Hôpital Ambroise Paré, Assistance Publique-Hôpitaux de Paris, Boulogne-Billancourt, France; 5 Department of Anesthesiology Cancer, University Institute of Toulouse-Oncopole, Toulouse, France; 6 Department of Medical Oncology, Centre Hospitalo-Universitaire Bretonneau, Tours, France; 7 University Federation of Radiation Oncology of Mediterranean Occitanie, Montpellier Cancer Institute, Université de Montpellier, Montpellier, France; 8 Department of Clinical Hematology and Cellular Therapy, Clermont-Ferrand University Hospital Center, Clermont-Ferrand, France; 9 Cancer Resistance Exploring and Targeting. EA7283, INSERM CIC501, BP 10448, Center, Clermont-Ferrand, France; 10 Department of Supportive and Palliative Care, Institut Curie, Paris, France; 11 Service D’oncologie Médicale Et de Thérapie Cellulaire, Pôle Onco-Hématologie, APHP—HôpitauxUniversitaires de L’estParisien, 75020 Paris, France; 12 Medical and Surgical Intensive Care Unit, Groupe Hospitalier Paris Saint Joseph, 185 Rue R. Losserand, 75674 Paris, France; 13 REQUIEM (Research/Reflexion On End of Life Support Quality in Everyday Medical Practice) Study Group, Paris, France

**Keywords:** Ethics, Advance directives, Cancer, Tumor, Oncologist, Trust person

## Abstract

**Background:**

The often poor prognosis associated with cancer necessitates empowering patients to express their care preferences. Yet, the prevalence of Advance Directives (AD) among oncology patients remains low. This study investigated oncologists' perspectives on the interests and challenges associated with implementing AD.

**Methods:**

A French national online survey targeting hospital-based oncologists explored five areas: AD information, writing support, AD usage, personal perceptions of AD's importance, and respondent's profile. The primary outcome was to assess how frequently oncologists provide patients with information about AD in daily clinical practice. Additionally, we examined factors related to delivering information on AD.

**Results:**

Of the 410 oncologists (50%) who responded to the survey, 75% (*n* = 308) deemed AD relevant. While 36% (*n* = 149) regularly inform patients about AD, 25% (*n* = 102) remain skeptical about AD. Among the respondents who do not consistently discuss AD, the most common reason given is the belief that AD may induce anxiety (*n* = 211/353; 60%). Of all respondents, 90% (*n* = 367) believe patients require specific information to draft relevant AD. Physicians with experience in palliative care were more likely to discuss AD (43% vs 32.3%, *p* = 0.027). Previous experience in critical care was associated with higher levels of distrust towards AD (31.5% vs 18.8%, *p* = 0.003), and 68.5% (*n* = 281) of the respondents expressed that designating a “person of trust” would be more appropriate than utilizing AD.

**Conclusion:**

Despite the perceived relevance of AD, only a third of oncologists regularly apprise their patients about them. Significant uncertainty persists about the safety and relevance of AD**.**

**Supplementary Information:**

The online version contains supplementary material available at 10.1186/s12910-024-01046-8.

## Introduction

Despite the recent tremendous progress in cancer care, cancerous diseases are still associated with a poor prognosis, with only 25% five-year survival after diagnosis [1]. Due to the immunologic dysfunction and frailty associated with cancer as well as treatment side effects [2], 15% of patients living with cancer will require admission to an intensive care unit (ICU) during their lives [3]. However, critical care, with a mortality rate up to 58%, poses a significant burden on patients. Not only is ICU care associated with notable patient discomfort [4], but survivors also encounter multiple challenges. These include increased mortality rate, diminished quality of life (with up to 50% of patients experiencing depression after critical care hospitalization [5] and 20% suffering from post-traumatic stress syndrome [6]), and altered autonomy (with only 50% of patients able to resume their previous activities five years after receiving critical care) [7]. It may also create unrealistic expectations among their relatives [4]. This significant impact on patients’ recovery, quality of life, and potential sequelae after critical care underscores the importance of understanding what is or is not acceptable for each individual patient. This understanding enables the delivery of the most appropriate and tailored care possible.

While a patient's prognosis and physiological reserve are often known and taken into account when determining ICU admission, it is common for patients in need of critical care to be unable to express their care preferences. To address this problem, Advance Directives (AD) were created, allowing patients to have a voice in their treatment decisions under all circumstances. The concept of AD was originally proposed and publicly introduced by Louis Kutner in the Indiana Law Journal in 1969 [8]. It was later legally implemented in the United States with the Patient Self-Determination Act in 1990. In Europe, this concept was adopted into the legal framework during the Oviedo Convention in 1997 and was subsequently recognized in France through the *Loi Leonetti* [9]. Current French law defines AD as a means for patients to express their wishes regarding the intensity of care, potential refusal of medical or surgical interventions, and their end-of-life care preferences [10]. By facilitating written, anticipatory expression of living wills by patients, AD serve as a crucial document in critical and life-threatening situations that necessitate swift, ethical decision-making. While AD have been part of the legal framework of patient care since 2005, have been medically binding since 2016, and have been promoted nationally by a specialized agency for end-of-life care (Centre National de la Fin de Vie et des Soins Palliatifs), fewer than 5% of French patients living with cancer have drafted their AD [11, 12]. Although factors such as a lack of information and the difficulties patients encounter in envisioning future circumstances [13] may contribute to the sparse use of AD, we investigated the possibility that medical hesitancy in applying these provisions may be another factor in the underutilization of AD. The main objective of this study was to assess how frequently information about AD is provided to patients in the daily clinical practice of oncologists. The secondary objectives focused on understanding the barriers to implementing AD within this context.

## Methods

### Study design

This study is a prospective, observational, cross-sectional study. It is based on volunteer participation and adheres to the applicable CROSS guidelines [14].

### Study population

All oncologists who were working full-time or part-time in a hospital setting at the initiation of the study were contacted to participate, including those at teaching hospitals, tertiary care hospitals, and cancer centers (the full list can be found in the Supplementary materials). Oncologists who worked exclusively in a private practice setting, physicians from non-oncological specialties, and resident physicians were excluded from the study.

### Precautions

No judgment was made regarding the use, compliance, or, conversely, the lack of application of AD — including AD information dissemination, documentation, and implementation. Details of the precautions taken during the study are described in the Supplementary materials.

### Questionnaire

The questionnaire was built according to the redaction guidelines for this type of instrument [15–18]. The various themes included in the questionnaire were selected using a Delphi procedure. The experts of the REQUIEM group determined which themes to include and proposed an initial draft of the questionnaire based on literature and critical elements identified in previous work [19–24]. This draft was then reviewed by experts from the French Society of Cancerology (*SFC: Société Française de Cancérologie*) and the French Society of bone marrow transplant and cellular therapy (*SFGM-TC: Société Francophone de Greffe de moelle et de Thérapie cellulaire*).

Five domains were chosen to structure a 19-item questionnaire: clinicians’ engagement in providing information about the existence of AD, clinicians’ involvement in writing AD, utilization of existing AD in case of an acute situation, information about the respondent’s department, and personal data regarding the respondent. The questionnaire was then pretested by various members of the REQUIEM group, consisting of intensivists, palliative care physicians, general practitioners, and pharmacists. However, we did not conduct a pilot study to avoid potential loss of respondents. The complete questionnaire is available in Appendix 1.

### Questionnaire administration

In June 2020 a center-specific link was generated and disseminated to respondents via email to access the electronic survey, which was stored and administered using Googleform®. We sent several reminders via telephone or email to the attending physicians across all the centers from mid 2020 to mid 2021. Centers were also notified of their response rate in comparison to other centers. The questionnaire was closed in June 2021.

### Study outcomes

The primary outcome was to assess the frequency at which information about AD is provided to patients in the daily clinical practice of oncologists. Secondary outcomes were factors influencing the provision of information about AD, assistance with AD drafting, physicians’ actual use of AD, possible means of improving the usage of AD, including the information needed to draft them according to the clinician’s experience, and the potential role of the “person of trust” (see definition in Supplementary materials).

### Statistical analysis

Data synthesis is presented as mean values and standard deviations (SD) or medians and interquartile ranges (IQR). The relationship between two qualitative parameters was evaluated using the Chi-square test. Differences were deemed significant if the alpha risk of identifying a non-existent difference was less than 5% (*p* ≤ 0.05).

### Ethics approval and consent to participate

The study was conducted in accordance with the principles laid out in the Declaration of Helsinki. This research falls under the French reference methodology MR003, and ethical approval was obtained from an ethics committee (*Groupe Ethique & Rercherche Médicale*—*GERM*. IRB 00012157). According to MR003 this type of research does not require specific consent (“*Recherches dans le domaine de la santé sans recueil du consentement*” Official text. Delibération n° 2018–154 of May 3, 2018; text n°109 of the *Journal officiel de la République française*). In this scenario, the ethics committee did not require a formal consent from participants. The respondents were informed about the study in writing, via an introductory paragraph at the beginning of the questionnaire. If a respondent did not reply to the questionnaire, it was considered as a refusal to participate in the study. On the other hand, completion of the questionnaire constituted de facto agreement to participate in the study.

### Consent for publication

Not applicable.

## Results

### Population

**Fig. 1:**
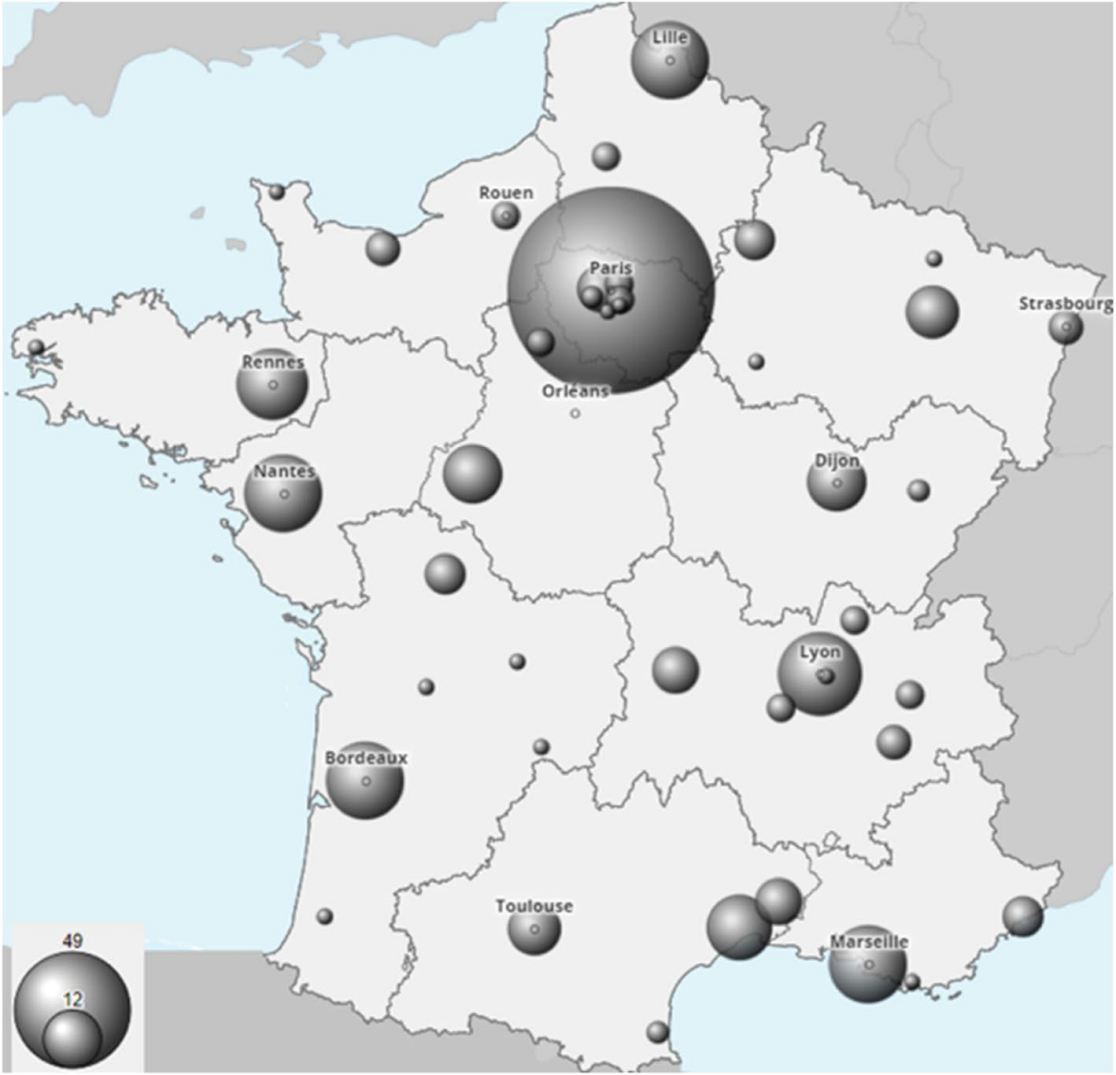
Geographical distribution of respondents

Between June 2020 and June 2021, 410 out of 818 oncologists who met the study criteria responded to the questionnaire. This represents a 50% response rate from oncologists working in 60 hospitals across France (Fig. 1). The respondents consisted of fellows (*n* = 101, 24.6%), attending physicians (*n* = 270, 65.9%) and professors (*n* = 39, 9.5%). This distribution accurately reflects the medical demographics in France. Of the respondents, 234 physicians (57.1%) work in cancer centers and 152 (37.1%) work in teaching hospitals (details in Table 1).

**Table 1:** Demographic caracteristics of respondents

Caracteristics		*N* = 410
Number of years of clinical practice		11 [6–20]
**Status**	Fellow	101 (24,6)
	Attending	270 (65,9)
	Professor	39 (9,5)
**Hospital type**	University hospital	152 (37,1)
	Cancer center	234 (57,1)
	Tertiary care hospital	23 (5,6)
Critical care experience		197 (48,0)
Palliative care experience		153 (37,3)

Values are expressed as median [IQR] or mean (%)

### Reported frequency of information provided to patients on Advance Directives (AD)

**Fig. 2:**
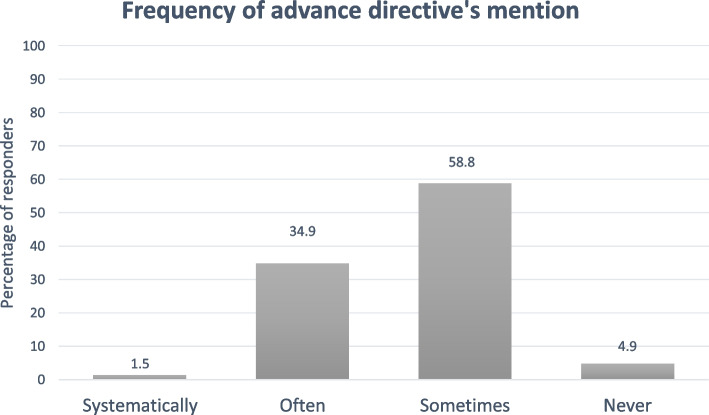
Frequency of Advance directive’s mention

Based on the responses, 36.3% (*n* = 149) of oncologists “often” or “systematically” discuss AD with their patients, while 59% (*n* = 241) do so “sometimes”. Of the respondents, 5% (*n* = 20) reported never discussing AD with their patients. These results are in Fig. 2.

### Factors Influencing the Provision of Information on AD

Demographic factors associated with more frequent discussions on AD include experience in palliative care (*p* = 0.027), working at a cancer center (*p* = 0.022), and increased professional experience (e.g., attending physicians are significantly more likely to discuss AD than fellows, *p* = 0.021) (see Appendix 3).

**Fig. 3:**
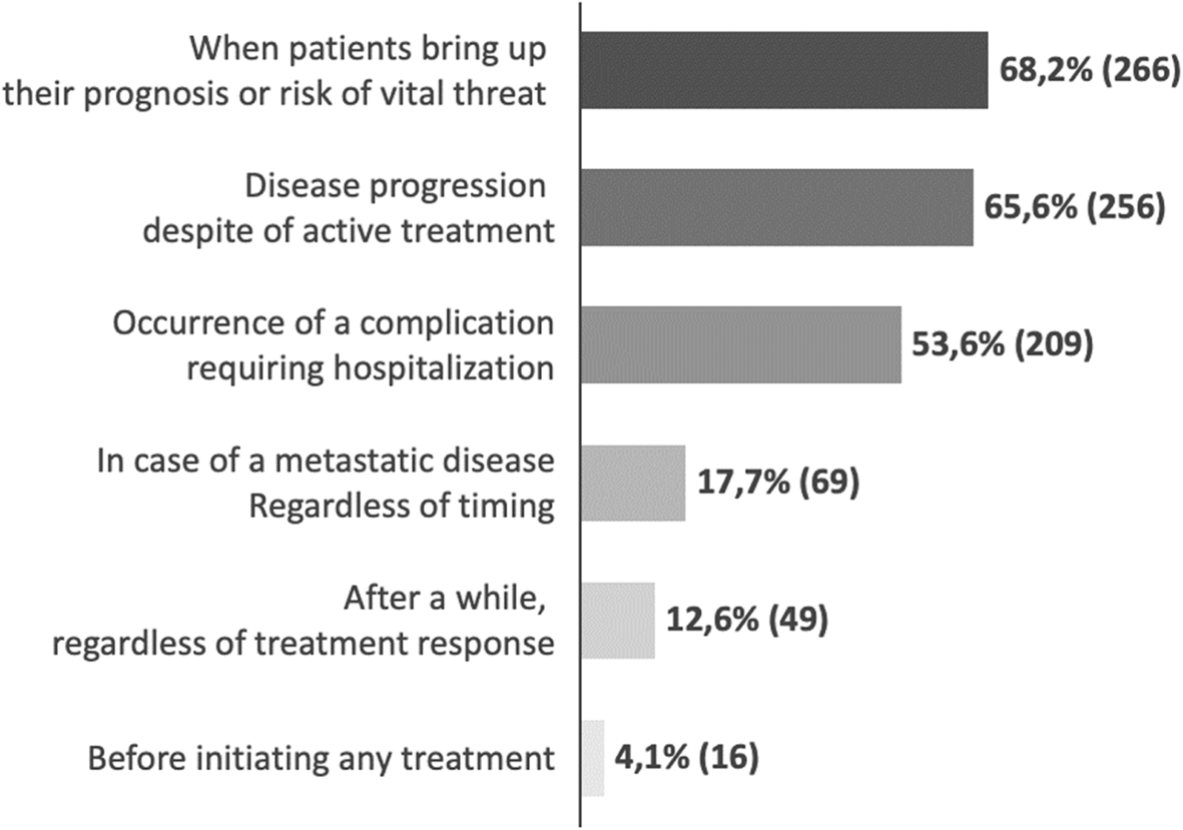
Timing for initiating a discussion on advance directives. Numbers are expressed in % (absolute value) of respondents to the survey. Respondents were allowed to choose multiple answers

Among the respondents who discuss AD with their patients, the main triggers driving these discussions include a patient’s questions about prognosis or vital risk, seen as a favorable context for 68.2% (*n* = 266) of the respondents. Other triggers include disease progression despite active treatment (*n* = 256; 65.6%), the occurrence of a complication requiring hospitalization (*n* = 209; 53%) and the presence of metastatic disease (*n* = 69; 17.7%) (Fig. 3).

**Fig. 4:**
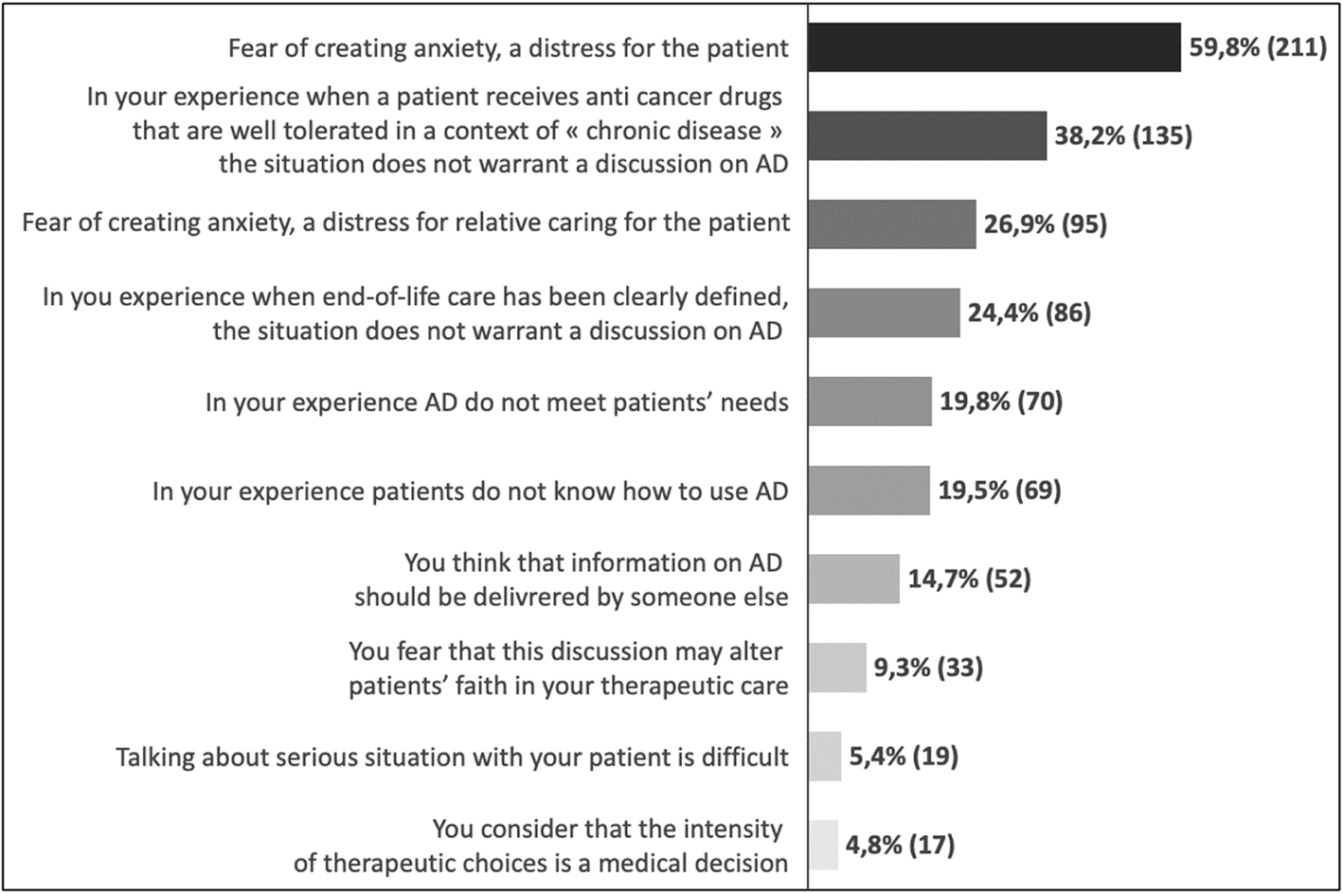
Reasons to avoid talking advance directives with a patient. Numbers are expressed in % (absolute value) of respondents to the survey. Respondents were allowed to choose multiple answers

Oncologists who do not routinely discuss AD often cite their concern about causing anxiety for the patient (*n* = 211; 59.8%) or their relatives (*n* = 95; 26.9%) as a primary reason. Other significant reasons include the perception that AD are not suitable for a chronic medical situation (*n* = 135; 38.2%), for end-of-life care (*n* = 86; 24.4%), or for meeting the overall needs of patients (*n* = 70; 19.8%), and 69 respondents (19.5%) voiced concern that patients may not know how to utilize AD (details in Fig. 4).

**Fig. 5:**
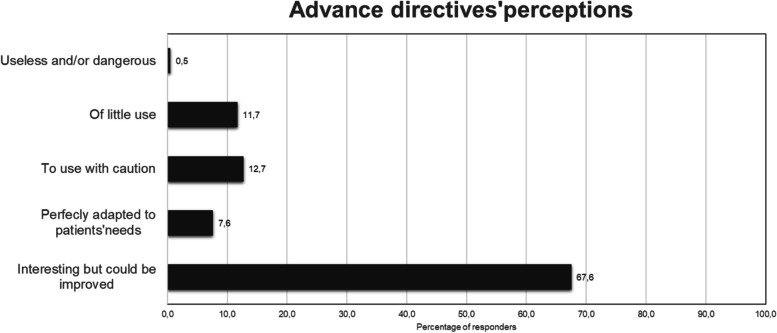
Oncologists perception of advance directives

There may also be a reluctance to use AD due to their perceived potential harmful implications. Indeed, 25% (*n* = 102) of the respondents express significant concerns regarding AD. Only 31 respondents (7.6%) believe that AD are perfectly suited to cover patients’ needs (Fig. 5). Furthermore, 56.8% (*n* = 233) of respondents feel that the responsibility to provide information on AD should lie with someone else, such as the primary care physician, state agency, or another entity (Appendix 2).

### Use of AD in clinical practice

Of the respondents, 32% (*n* = 130) have assisted their patients in drafting their AD. Physicians who often discuss AD were more likely to have aided their patients in this process (*p* = 0.0005) (Appendix 4). Additionally, 50.9% (*n* = 209) of respondents stated that they collect AD and 46.8% (*n* = 192) said that they formally collect decisions regarding treatment limitations. When respondents had access to AD, 90.2% (*n* = 370) reported adhering to them. 9.8% (*n* = 40) clinicians said they sometimes disregarded AD. Within this subgroup, the reasons cited for not respecting the AD were urgent situations that fell outside the scope of patients’ end-of-life care (87%, *n* = 35) or concern that the patient may have changed their mind (35.0%, *n* = 14).

### Potential improvement of AD usage

**Fig. 6:**
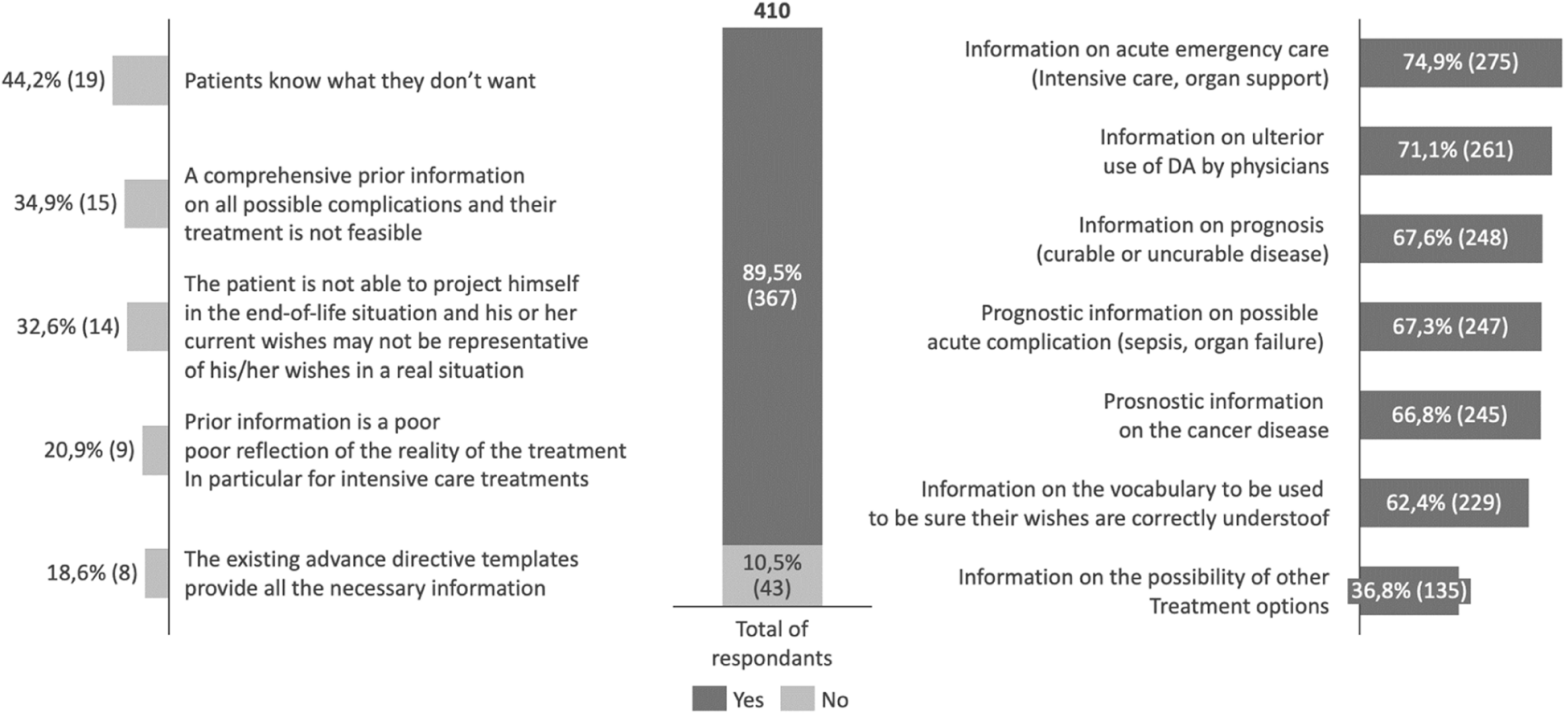
Necessity and type of additional information on AD. Numbers are expressed in % (absolute value) of respondents to the survey. Respondents were allowed to choose multiple answers

Although currently underutilized, 75.1% (*n* = 308) of respondents perceive AD as a valuable tool. In fact, 89.5% (*n* = 367) of the responding oncologist believe that patients require additional information to effectively write their AD. Additional information they highlighted include information on their general care (*n* = 275; 74.5%), their prognosis (*n* = 248, 67.6%), available treatments (*n* = 135, 36.8%), how to use AD (*n* = 261; 71.1%), and specific medical terminology (*n* = 229; 62.4%) (Fig. 6).

### Person of trust

Systematic recording and tracking of information about the person of trust designated by the patient was reported by 86.1% (*n* = 350) of respondents, and 73.6% (*n* = 302) acknowledged having a specific section in their medical records dedicated for documenting this information.

Of respondents, 52.4% (*n* = 215) “systematically” discuss the concept of the person of trust with their patients, while only 19% (*n* = 80) “rarely” or “never” broach the topic. Furthermore, 68.5% (*n* = 281) of the respondents believe that designating a trust person would be a more effective tool than AD for managing patients’ care. This sentiment was notably more prevalent among younger physicians (Appendix 5).

## Discussion

In our national survey, we found that 36% of respondents regularly, if not systematically, discuss AD with their patients. The topic of AD is more likely to be addressed when patients express a desire for information about prognosis or vital risk or when their condition deteriorates. On the other hand, the trepidation of creating anxiety for the patient or their relatives, especially in the case of a stable and chronic disease, is a significant factor deterring the mention of AD. Ultimately, oncologists regard the designation of a person of trust as an important means for conveying patients’ wishes.

This study highlights a significant challenge in the use of AD: the ambivalence felt by clinicians. While 47% of respondents see AD as a means to reassure their patients, the predominant reason cited for not discussing AD is the fear of causing distress. This fear, [25] as well as the concern about impacting the therapeutic relationship [26] are often noted in the literature. However, a large multicenter study found discussing prognosis, including the progression of tumor diseases and end-of-life scenarios did not cause harmful effects [27]. In fact, early discussion of advance care plans may even provide psychological benefits and lessen the risk of unwanted aggressive care [27–29], which has been shown to negatively impact patients’ quality of life [27, 28, 30, 31].

The timing of discussing AD represents a significant challenge for physicians. Our study revealed the majority of oncologists consider it preferable to wait for a favorable context, typically marked by patient’s inquiries about their disease prognosis or the vital risk associated with the tumor. However, the vast majority of patients seem to prefer that their physicians initiate such discussions rather than the patients themselves [26, 32–34]. While the most appropriate timeframe for these discussion is still uncertain, recent studies have confirmed the feasibility of systematically informing patients with severe tumor diseases about AD at an early stage [35, 36].

While the legislative framework [37, 38] and educational training [39] of medical students have evolved over recent decades to prioritize patient autonomy in care processes, young clinicians often remain hesitant to discuss AD with their patients. This trend is evidenced by a study involving young general practitioners; despite broadly supporting the implementation of the provision, they only considered malignant disease diagnosis as an opportunity to inform their patients in 60% of cases [23]. These findings underscore the importance of developing training programs that include comprehensive information on the safety and benefits of initiating discussions about AD [32, 40]. Simulation-based communication-training programs may prove effective for this purpose [41, 42]. Additionally, a potential strategy for enhancing the implementation of AD could involve systematically providing information on AD existence and collection during the initial management of patients, regardless of disease severity [43].

To overcome the challenges associated with AD, 68.5% of the respondents believe that designating a person of trust would be more applicable for patient care due to its dynamic nature. The role of a person of trust appears to be favored by patients as well. In two independent studies (HELP [44] and SUPPORT [45]) conducted among elderly and cancer patients, 70% and 78% of respondents respectively were comfortable with their loved ones making medical decisions on their behalf if they were to become incapacitated [46]. However, patients seldom discuss their preferences for medical care, particularly in hypothetical critical and severe scenarios, with their relatives or persons of trust. The agreement between a patient's decisions and those of their person of trust can be as low as 30% [44, 47, 48]. Similarly, both physicians and persons of trust seem incapable of accurately gauging what their patient or relative would consider as an acceptable quality of life [49]. The role of a person of trust is not without responsibilities. Involvement in the decision to escalate therapy can be a source of psychological distress for the patient's relatives, especially for the person of trust [50]. Furthermore, the person of trust may be more inclined to prioritize keeping their loved one alive, possibly at the expense of the patient’s comfort [51]. Therefore, if the person of trust is to become a primary participant in the care of an incapacitated patient, they should not replace AD, but rather function in conjunction with these directives.

Beyond the communication challenges related to AD, disease prognosis, or end-of-life discussions; our findings highlight a significant issue; clinicians often believe they can determine when a patient should record their own preferences regarding care intensity. This restriction of patient’s choice, illustrated by the absence of information about AD when the situation is medically deemed inappropriate or the presumed inadequacy of AD to meet patient needs, along with persistent failure to comply with existing AD, tend to suggest persistent medical paternalism. While patient autonomy is universally acknowledged as a central element of patient care, it is evident that numerous scenarios are still deemed as exceptions [52]. The capacity of patients to anticipate their wishes in the case of cognitive decline is a central point in this debate [53, 54], and similar issues come to the forefront during the management of cancer [33]. Additionally, a study involving over 4000 oncologists in the United-States revealed heightened hesitation to discuss care intensity or end-of-life preference, compared to non-oncologist physicians [33]. However, the lack of discussion regarding AD does not necessarily equate to an absence of dialogue about disease prognosis or patients' care preferences [33]. While our study is not equipped to address this particular query, the overall commitment demonstrated by the respondents could be interpreted as a desire to offer the best possible care to patients, regardless of the presence of AD. Similarly, the lack of introduction of AD and pre-planning of care may reflect i) the physician's uncertainty regarding the predictability of the disease's progression [54, 55], and ii) the perception that patients require more comprehensive information to make accurate decisions [53, 55], as illustrated in the *potential improvement section* of our work. Unfortunately, the demand for more comprehensive data regarding the disease's status may obscure the perceived inability of patients to determine their "best interest" [53, 56, 57], which is another manifestation of medical paternalism. These observations underscore the importance of clarifying the distinction between “medical expertise (which refers to specialized knowledge in a certain area) and “medical authority” (the assumed prerogative to make decisions based on that knowledge) [58].

This study does have limitations. For instance, the response rate is not entirely satisfactory. However, to our knowledge, this is the first and largest study of its kind conducted among French hospital oncologists. Furthermore, the representativeness of the study appears to be preserved given that responses are widely distributed across the country, and the distribution among fellows, attendings, and professors mirrors the real-life demographic spread.

Our survey was accessible at each center for a year. The unique link was closed as soon as all potential respondents completed the survey. This extended period may impact the results in different ways. First, the study may have stimulated discussions about AD among colleagues potentially leading to modifications in local practices related to this subject. Second, practices may have evolved over the course of a year in France owing to continuous improvements in clinical practice and a growing awareness of the challenges associated with end-of-life care, particularly in the context of the ongoing public debate about end-of-life decisions and euthanasia in the country. However, these factors could result in a potential over-reporting of AD use. Despite this, our current results still underscore potential strategies for enhancing the broader usage of AD.

Another limitation of the study is the recruitment bias. The oncologists who chose to respond to the questionnaire are likely those most invested in the concept of AD. Moreover, the proportion of responding practitioners with palliative care experience (37%) is greater than in the general population of French oncologists. Accordingly, these responses may skew toward being more supportive of AD compared to the general sentiment held by oncologists.

Lastly, as the responses are self-reported, there is not an absolute guarantee of their validity in day-to-day practice. Nevertheless, given the survey was anonymous and factual, without implying a need for practice change, there seem to be no distinct motives for respondents to misrepresent their views while completing the questionnaire.

## Conclusion

Three-quarters of oncologists believe that AD can be useful. However, only one-third of the oncologists regularly discuss them. The primary reasons for such hesitation encompass concerns about potentially generating or amplifying patient anxiety, as well as the perception that AD may not be suitable for the patient's specific medical circumstances.

### Supplementary Information

**Supplementary Material 1.**

**Supplementary Material 2.**

## Data Availability

Datasets used/analyzed in this study are accessible from the REQUIEM study group upon reasonable request.

## Abbreviations

AD: Advance Directives
ICU: Intensive Care Unit
IQR: Interquartile Range
SD: Standard deviation
