# Supplementary material for: Oncologists’ perspective on advance directives, a French national prospective cross-sectional survey – the ADORE study
Source: BMC Med Ethics. 2024 Apr 10;25:44. doi: 10.1186/s12910-024-01046-8 (PMC11008039; doi:10.1186/s12910-024-01046-8)
Supplement: Supplementary file 1 — Supplementary Material 1. [file 12910_2024_1046_MOESM1_ESM.docx]

Supplementary material

**Study population**

All oncologists working full -or partial- time in a hospital setting (teaching hospitals, tertiary hospitals, cancer centers; list in supplementary material) were included. *Of note, in France, most patients with cancer are treated in the network of cancer centers (“Unicancer, Centres de lutte contre le cancer”), that are non-profit private structures with an academic and teaching vocation, exclusively dedicated to cancer care.*

Exclusions criteria were: oncologists working exclusively in a liberal setting, or working exclusively in functions not immediately related to the specialty indicated; physicians from other specialties, residents.

**Precautions**

The rationale for the survey was based on the existence of difficulties illustrated by the lack of information, drafting and use of advance directives in the course of daily clinical activity. Therefore, no judgement was made on the use, respect or, on the contrary, the lack of application of advance directives (information, writing, use).

A written information specifying the anonymous and voluntary character of the study preceded the questionnaire. The anonymity of the response was particularly highlighted.

No mention was made regarding the possible need to modify the personal behavior of the clinicians interviewed, the question raised being rather that of improving the structuring of information and assistance in drafting advanced directives in addition to their own work. Thus, they may wish to take part or, on the contrary, to delegate these functions to another clinician (as the literature seems to indicate).

The absence of response to the questionnaire was consider secondary to a refusal to participate. Conversely, completion of the questionnaire constituted *de facto* acceptance of participation in the study.

**Person of trust**

The person of trust, different from a person with power of attorney, is a person that may be chosen by the patient to be the privileged person to receive medical information and discuss therapeutic options and report patient’s wishes with the caring team if the patient becomes unable to do so.
