# Supplementary material for: Oncologists’ perspective on advance directives, a French national prospective cross-sectional survey – the ADORE study
Source: BMC Med Ethics. 2024 Apr 10;25:44. doi: 10.1186/s12910-024-01046-8 (PMC11008039; doi:10.1186/s12910-024-01046-8)
Supplement: Supplementary file 2 — Supplementary Material 2. [file 12910_2024_1046_MOESM2_ESM.docx]

APPENDIX

Appendix 1A : Questionnaire – French Version

Chère consœur, cher confrère,

Le groupe de recherche clinique REQUIEM (www.grouperequiem.com), vous propose de participer à une étude nationale sur l’usage des directives anticipées en oncologie et hématologie.

La population de patients d’oncologie et d’hématologie est, du fait de sa particulière fragilité, exposée à un risque élevé de complications aiguës sévères, pouvant impliquer un trouble de vigilance. L’intensité thérapeutique dans ce contexte est soumise à la pertinence de la réanimation, elle-même sous tendue par le bénéfice attendu mais également par les souhaits éventuels du patient quant à la suppléance d’organes ou plus généralement à ses préférences de survie ou de confort.

Dans ces conditions les directives anticipées sont d’un apport certain, mais restent peu utilisées, notamment chez les patients d’oncologie et d’hématologie. Notre objectif est de mieux comprendre l’intérêt porté à ces directives par les Oncologues et Hématologues ainsi que les limites perçues à leur application pratique, afin de proposer dans un second temps des alternatives aux obstacles identifiés.

Votre participation nous est précieuse en cela qu’elle nous permettra de mieux comprendre la perception des directives anticipées par les cliniciens de terrain ainsi que les limites à leur usage et à leur mise en pratique.

Nous restons à votre disposition pour tout complément d’information et nous ferons un devoir de vous informer des résultats obtenus à la fin de cette étude.

Très confraternellement.

Les directives anticipées sont apparues en 2005 (loi Léonetti du 22 avril 2005) et ont été modifiées en 2016 (loi Claeys- Léonetti du 02 février 2016)

1. **Information des patients concernant la loi (directives anticipées et personne de confiance)**

- Vous suggérez à vos patients de désigner une personne de confiance
  - Systématiquement
  - Souvent
  - Parfois
  - Jamais
- Dans votre expérience des directives anticipées sont

| Parfaitement adaptées aux besoins des patients | Intéressantes mais pourraient être améliorées | Peu utiles | A utiliser avec beaucoup de précautions | Inutiles et ou dangereuses |
| --- | --- | --- | --- | --- |

- Vous arrive-t-il d’aborder le sujet des directives anticipées avec vos patients ?
  - Systématiquement
  - Souvent
  - Parfois
  - Jamais
- Si oui, à quel moment ? (NB question conditionnelle)
  - Avant l’initiation de tout traitement
  - Au bout d’un certain délai indépendamment de la réponse au traitement
  - Indépendamment du délai, en cas d’atteinte (multi)-métastatique
  - En cas d’évolutivité de la maladie malgré le traitement
  - En cas de dégradation ayant nécessité une hospitalisation
  - Lorsque le patient évoque le sujet du pronostic ou du risque vital
- Dans votre expérience, quel est l’intérêt de parler des directives anticipées avec vos patients ? (une ou plusieurs réponses)
  - Cela permet d’aborder les complications possibles de leur maladie
  - Cela vous permet de mieux connaître les désirs de vos patients concernant leur fin de vie
  - Cela permet de personnaliser la prise en charge et de rassurer les patients
  - Cela permet de faciliter la discussion et la prise en charge avec les différents intervenants (médecin généraliste, spécialiste d’organe, réanimateur…)
  - Vous ne parlez pas des directives anticipées avec vos patients
  - Autre (précisez) :
- Si vous n’en parlez jamais avec vos patients c’est parce que : (une ou plusieurs réponses) (NB question conditionnelle)
  - Vous craignez de créer de l’angoisse chez votre patient
  - Vous craignez que cette discussion altère la confiance du patient dans votre prise en charge thérapeutique
  - Dans votre expérience les patients ne savent pas faire usage des directives anticipées
  - Parler des situations graves avec votre patient vous est pénible
  - Vous considérez que l’intensité thérapeutique est une décision médicale
  - Dans votre expérience les directives anticipées ne sont pas adaptées aux besoins des patients
  - Vous considérez que l’information sur l’existence des DA devrait être réalisée par un autre intervenant, plutôt que l’oncologue / hématologue
- Si vous n’en parlez pas systématiquement selon les patients c’est parce que: (une ou plusieurs réponses)  (NB question conditionnelle)
  - Vous craignez de créer de l’angoisse, une détresse chez votre patient
  - Vous craignez de créer de l’angoisse, une détresse pour le proche qui l’accompagne
  - Vous craignez que cette discussion altère la confiance du patient dans votre prise en charge thérapeutique
  - Dans votre expérience, quand le patient reçoit un traitement anti-cancéreux efficace et bien toléré en situation de maladie chronique situation médicale ne s’y prête pas
  - Dans votre expérience quand le projet de fin de vie a été clairement élaboré la situation médicale ne s’y prête pas.
- Dans votre expérience quel intervenant devrait réaliser prioritairement l’information sur l’existence des directives anticipées :
  - Oncologue / Hématologue
  - Médecin traitant
  - Pharmaciens d’officine
  - Pouvoirs publics (courrier, campagnes publicitaires, journées spécifiques…)
  - Autre : précisez :

1. **Assistance à la rédaction des DA**

- Si oui : A partir de votre expérience clinique, de quel(s) type(s) d’information a besoin le patient pour rédiger ses directives anticipées ? (une ou plusieurs réponses)
  - Informations pronostiques sur sa maladie tumorale
  - Informations pronostiques sur les maladies aiguës qui peuvent survenir (choc septique, défaillance d’organe…)
  - Informations sur la prise en charge en urgence de ces maladies aiguës (réanimation, suppléances d’organes)
  - Informations sur les termes à employer pour que leurs volontés soient bien comprises
  - Informations sur l’usage ultérieur des DA par les médecins
  - D’informations concernant l’existence d’éventuelles autres lignes de traitements efficaces sur leur maladie tumorale (voire du nombre de ces lignes)
  - De la notion de curabilité ou d’incurabilité de la maladie, même si les délais sont de détermination délicate.
  - Aucune information spécifique
- Si non, pourquoi ? (une ou plusieurs réponses) :
  - Les formats de DA existants proposent toutes les informations nécessaires
  - Les patients savent ce qu’ils ne souhaitent pas, indépendamment du pronostic de la maladie ou de la technicité spécifique des suppléances d’organes
  - Le patient n’est pas capable de se projeter dans la situation de fin de vie et ses volontés éventuelles risquent d’être peu représentatives de ses volontés en situation réelle.
  - L’information préalable est un mauvais reflet de la réalité du traitement (notamment en réanimation)
  - Une information préalable exhaustive sur toutes les complications possibles et leurs traitements n’est pas réalisable
- Vous arrive-t-il d’aider vos patients à rédiger leurs directives anticipées ? :
  - Oui
  - Non
- Si oui, vous proposez votre aide (une ou plusieurs réponses) (NB : question conditionnelle):
  - Vous proposez spontanément votre aide
  - Vous répondez favorablement aux patients qui vous sollicitent
  - Vous n’acceptez que si vous pensez qu’ils/elles comprennent les implications de leurs DA

1. **Usage des directives anticipées**

- Considérant vos patients qui ont rédigé des directives anticipées : vous est-il arrivé de ne pas en tenir compte dans l’évaluation de l’intensité des soins et/ou la mise en œuvre de limitations thérapeutiques :
  - Oui
  - Non
- Si vous n’en avez pas tenu compte, pourquoi (une ou plusieurs réponses) ? :
  - La situation urgente s’est présentée alors que le patient n’était pas dans la situation médicale de fin de vie
  - Vous pensez que vous risquez d’avoir des ennuis avec la justice si tous les moyens thérapeutiques n’ont pas été mis en œuvre
  - La famille ou l’entourage du patient ne sont pas d’accord avec les décisions inscrites dans les DA
  - Vous craignez que le patient n’ait changé d’avis depuis qu’il a rédigé les DA
  - Selon votre expérience clinique, une personne de confiance (ou une personne équivalente ne correspondant pas à la définition précise de la PC) bien informée des souhaits du patient pourrait, en permettant une **discussion dynamique** ?
  - Oui
  - Non

1. **Informations concernant le service**

- Dans les dossiers médicaux des patients existe-t-il :
- Une traçabilité systématique de l’information sur la personne de confiance ?
- Un lieu dédié pour le recueil de la personne de confiance si elle est désignée par le patient ?
- Un lieu dédié pour le recueil des directives anticipées si elles sont rédigés par le patient ?
- Un recueil formalisé des décisions de limitation thérapeutiques ?
- Aucune des informations ci-dessus n’est systématiquement recueillie ?

1. **Informations concernant le répondant**

- Êtes-vous déjà passé dans un service de réanimation en tant qu’interne ?
  - Oui
  - Non
- Êtes-vous déjà passé dans un service de soins palliatifs en tant qu’interne ?
  - Oui
  - Non
- Vous exercez en Oncologie depuis : ______ ans
- Dans ce service vous êtes :
- PUPH
- PH ou équivalent
- CCA, assistant, PH contractuel

Appendix 1B: questionnaire (translated in English)

Dear colleague,

The REQUIEM clinical research group (www.grouperequiem.com) invites you to participate in a national study on the use of advance directives in oncology and hematology.

The oncology and hematology patient population is, due to its particular frailty, exposed to a high risk of severe acute complications, which may involve a loss of vigilance. The intensity of therapy in this context is subject to the relevance of resuscitation, which is itself underpinned by the expected benefit but also by the patient's possible wishes regarding organ replacement or, more generally, his or her preferences for survival or comfort.

Under these conditions, advance directives are of definite help, but remain little used, especially in oncology and hematology patients. Our objective is to better understand the interest of oncologists and hematologists in these directives as well as the perceived limits to their practical application, in order to propose alternatives to the identified obstacles.

Your participation is valuable to us as it will allow us to better understand the perception of advance directives by clinicians in the field as well as the limits to their use and implementation.

We remain at your disposal for any further information and will inform you of the results obtained at the end of this study.

Very confraternally.

Advance directives appeared in 2005 (Léonetti law of 22 April 2005) and were modified in 2016 (Claeys- Léonetti law of 02 February 2016)

1. Information to patients regarding the law (advance directives and trusted person)

- You suggest to your patients to designate a trust person
  - Systematically
  - Often
  - Sometimes
  - Never
- In you experience, Advance directives are:
  - Perfectly adapted to patient’s needs
  - Interesting but could be improved
  - To be used with caution
  - Of little use
  - Useless and/or dangerous
- Do you ever discuss advance directives with your patients?
  - Systematically
  - Often
  - Sometimes
  - Never
- If so, in which timing? (NB conditional question) (one or more answer)
  - Before the initiation of any treatment
  - After a certain time independently of treatment response
  - Independently of timing, when disease is (multi)-metastatic
  - In case of progression despite treatment
  - In case of clinical deterioration requiring hospitalization
  - When the patient talks about prognosis or vital risk.
- In your experience, what is the value of discussing advance directives with your patients? (One or more answers)
  - It allows you to discuss the possible complications of their disease
  - It allows you to better understand your patients' wishes regarding their end of life
  - It allows you to personalize care and reassure patients
  - It facilitates discussion and management with the various parties involved (general practitioner, organ specialist, resuscitator, etc.)
  - You do not discuss advance directives with your patients
  - Other (specify):
- If you don't talk about AD (never or not systematically) to your patients it is because: (one or more answers):
  - You are afraid of creating anxiety or distress for your patient
  - You are afraid of creating anxiety or distress for the person accompanying him/her
  - Talking about serious situations with your patient is painful for you
  - You are afraid that this discussion will alter the patient's confidence in your therapeutic management
  - In your experience, when the patient is receiving effective and well-tolerated anti-cancer treatment in a "chronic disease" situation, the conditions do not lend themselves to it
  - You consider that therapeutic intensity is a medical decision
  - In your experience, when the end-of-life project has been clearly elaborated, the medical situation does not lend itself to it.
  - In your experience, patients do not know how to use advance directives
  - In your experience, advance directives are not adapted to the patient's needs
  - You consider that information on the existence of ADs should be provided by someone else, rather than the oncologist/hematologist
- In your experience, who should be the first to provide information about the existence of advance directives?
  - Oncologist
  - Primary care physician
  - Pharmacist
  - Public authorities (mail, advertising campaigns, specific days...)
  - Other (specify):

1. Assistance in the drafting of AD

- In your experience, do patients need specific information (through a written document or medical information) to be able to draft their AD?
  - Yes
  - No
- If yes: Based on your clinical experience, what type(s) of information does the patient need to write advance directives? (One or more answers)
  - Prognostic information about his tumor disease
  - Prognostic information on acute diseases that may occur (septic shock, organ failure...)
  - Information on the emergency management of these acute diseases (resuscitation, organ replacement)
  - Information on the terms to be used so that their wishes are well understood
  - Information on the subsequent use of AD by physicians
  - Information about the existence of other effective lines of treatment for their tumor disease (or the number of such lines)
  - The notion of curability or incurability of the disease, even if the time frame is difficult to determine.
- If not, why? (One or more answers)
  - Existing AD formats offer all the necessary information
  - Patients know what they do not want, regardless of the prognosis of the disease or the specific technicality of organ replacement
  - Patients are not able to project themselves into the end-of-life situation and their wishes may not be representative of their real-life wishes.
  - Prior information is a poor reflection of the reality of the treatment (especially in intensive care)
  - It is not possible to provide exhaustive advance information on all possible complications and their treatment
- Do you ever help your patients write their advance directives?:
  - Yes
  - No
- If yes, you offer your help (one or more answers) (NB: conditional question):
  - You spontaneously offer your help
  - You respond favorably to patients who ask for your help
  - You accept only if you think they understand the implications of their Ads

1. Use of advance directives

- Considering your patients who wrote advance directives: have you ever disregarded them when assessing the intensity of care and/or implementing therapeutic limitations?
  - Yes
  - No
- If you disregarded them, why? (one or more answer)
  - The emergency situation arose when the patient was not in a medical situation of end of life
  - You feel you may be in trouble with the law if all therapeutic means have not been used
  - The patient's family or friends do not agree with the decisions stated in the advance directive
  - You are concerned that the patient has changed his or her mind since he wrote his AD
- In your clinical experience, a trust person (or equivalent person not corresponding to the precise definition of TP) well informed of the patient's wishes could, by allowing a dynamic discussion (in time but also according to the different potential episodes), be more suitable than advance directives (which are static and rigid)?
  - No
  - Yes

1. Information about the department

- In patients’ medical file, is there?
  - Systematic traceability of information on the trust person?
  - A dedicated place for the collection of the trust person if designated by the patient?
  - A dedicated place for the collection of advance directives if they are written by the patient?
  - A formalized record of decisions on therapeutic limitations?
  - None of the above information is systematically collected?

1. Information about the respondent

- Have you ever had a critical care experience during your residency?
  - No
  - Yes
- Have you ever had a palliative care experience during your residency?
  - No
  - Yes
- You have been an oncologist for ….. years
- In this department you are
  - Professor
  - Attending
  - Fellow


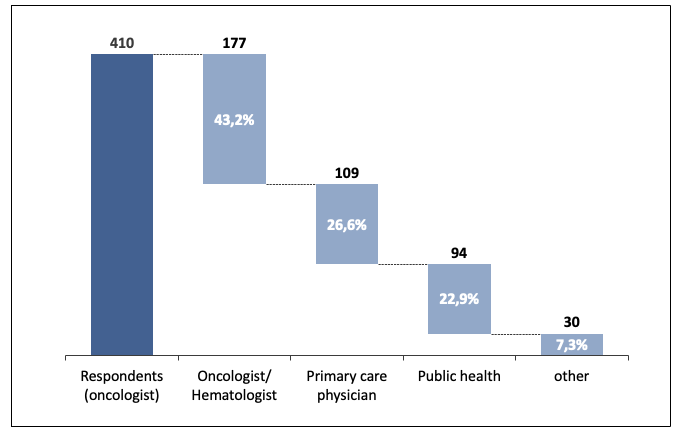


Appendix 2 : Stakeholder of Choice for Information on Advance Directives


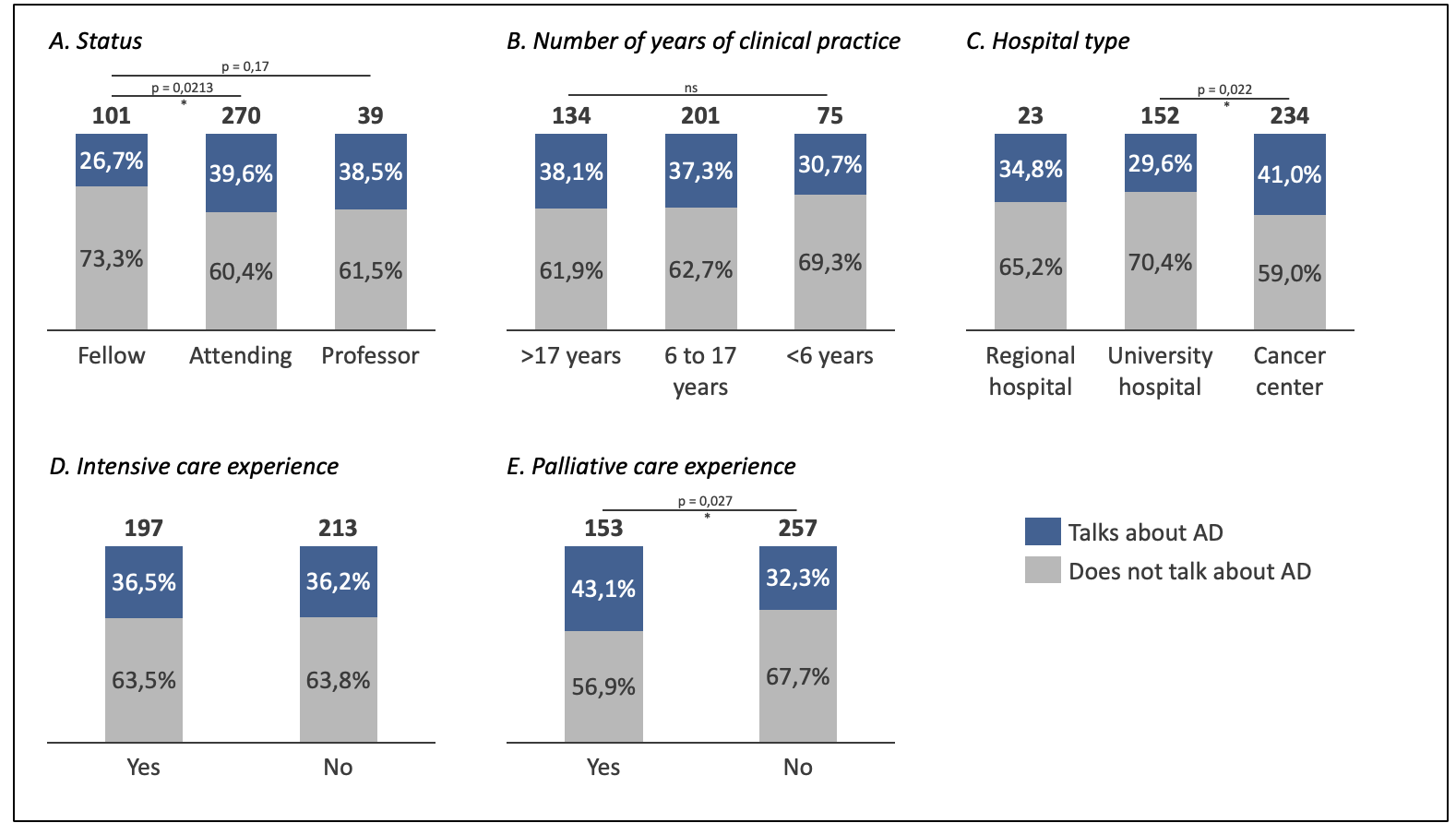


Appendix 3: Percentage of physicians talking about AD or not according to A. their hierarchical rank, B. their number of year of clinical practice (6 and 17 years breakdown have been chosen because they correspond to the implementation of the 2 French laws on end-of-life care and advance directives), C. the hospital type, D. clinicians previous experience in intensive care, E. clinicians previous experience in palliative care.

*Absolute numbers of respondents are shown on top of the bar graphs. Proportions of clinicians talking or not about AD are expressed in percentages.*


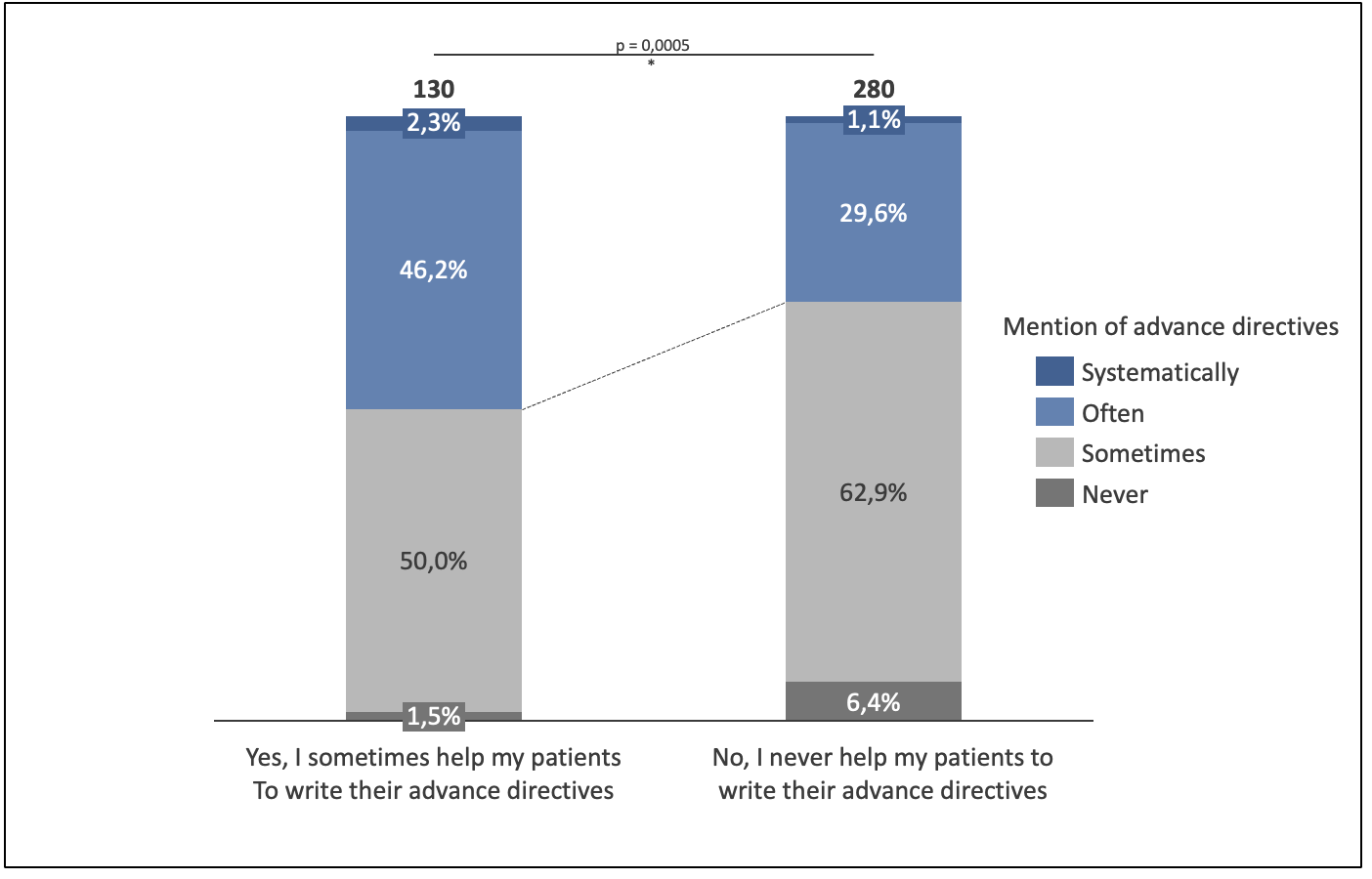


Appendix 4: Correlation between the mention of advance directives to patients and physicians willingness to help their patients in advance directive redaction.

*Absolute numbers of respondents are shown on top of the bar graphs. Proportions of clinicians talking or not about AD are expressed in percentages.*


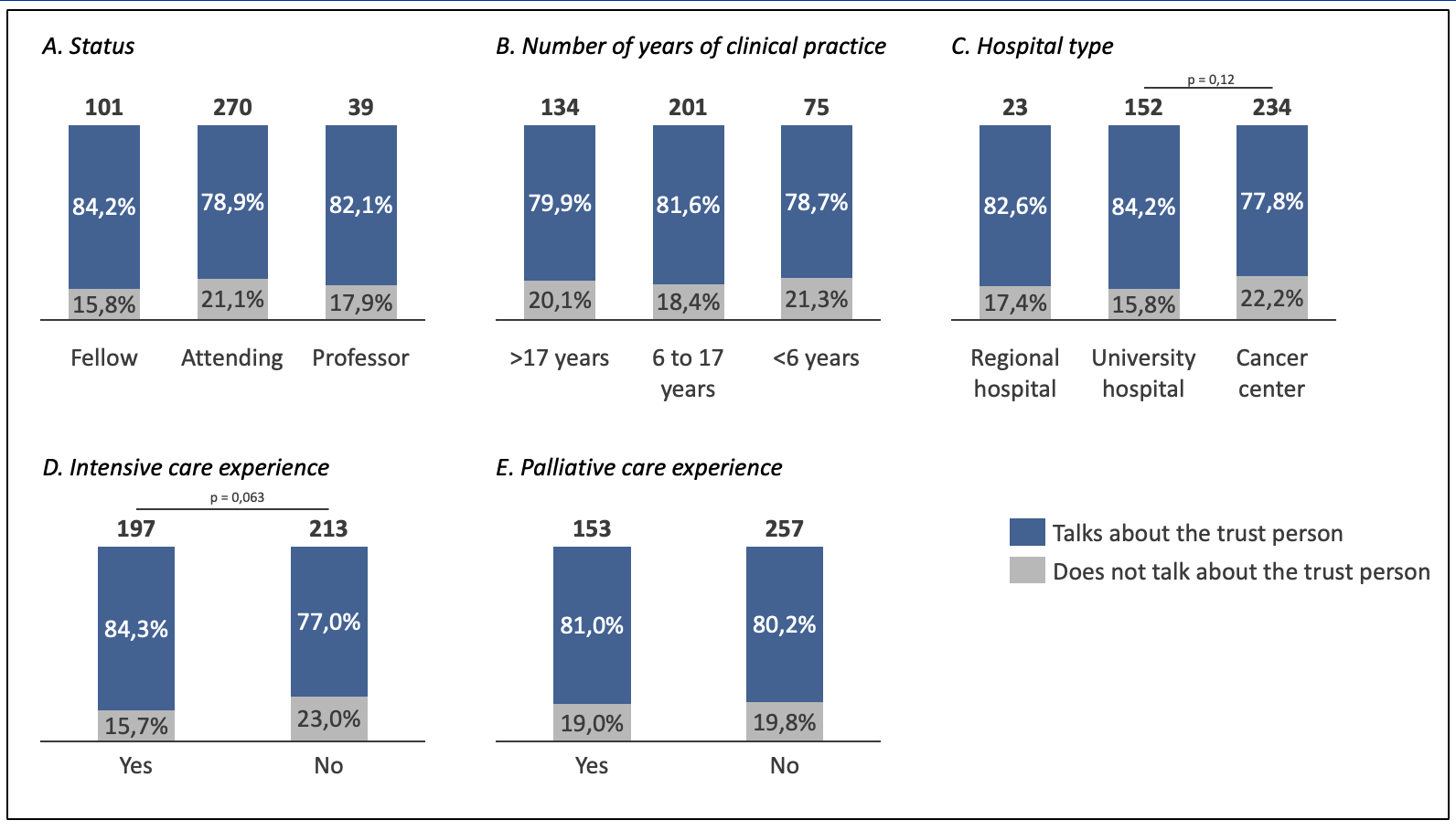


Appendix 5: Percentage of physicians who talk about the trust person according to A. their hierarchical rank, B. their number of year of clinical practice (6 and 17 years breakdown have been chosen because they correspond to the implementation of the 2 French laws on end-of-life care and advance directives), C. the hospital type, D. clinicians previous experience in intensive care, E. clinicians previous experience in palliative care.

*Absolute numbers of respondents are shown on top of the bar graphs. Proportions of clinicians talking or not about AD are expressed in percentages.*
